# Synthesis of Ce/Gd@HA/PLGA Scaffolds Contributing to Bone Repair and MRI Enhancement

**DOI:** 10.3389/fbioe.2022.834226

**Published:** 2022-03-31

**Authors:** Xianji Song, Xilin Liu, Yihang Ma, Qingsan Zhu, Mingchao Bi

**Affiliations:** ^1^ Department of Spine Surgery, China-Japan Union Hospital of Jilin University, Changchun, China; ^2^ Department of Hand Surgery, China-Japan Union Hospital of Jilin University, Changchun, China; ^3^ Department of Ophthalmology, The First Hospital of Jilin University, Changchun, China

**Keywords:** nanoparticles, co-doping, bone repair, MRI, cerium, gadolinium

## Abstract

It is important for future clinical applications to design and synthesize multipurpose scaffolding materials for bone tissue engineering with high osteogenic induction and MRI capability. In the present study, we synthesized Ce/Gd@HA by co-doping Ce^3+^ and Gd^3+^ into hydroxyapatite (HA) using a hydrothermal synthesis method, and then Ce/Gd@HA composites were synthesized by combining Ce/Gd@HA nanoparticles with polylactic-co-glycolic acid (PLGA) to investigate whether implanted Ce/Gd@HA/PLGA composites could promote osteoblast viability, leading to tibia repair of the rats and enhance MRI. The measurement results contain X-ray diffraction (XRD), Fourier-transform infrared (FTIR) spectroscopy, and environmental scanning electron microscopy (ESEM) showing that HA doped with Ce^3+^ and Gd^3+^ was still a hexagonal crystal with high crystallinity. The synthesized Ce/Gd@HA/PLGA composites have a structure and obvious magnetic resonance imaging (MRI) capability. The *in vitro* experimental results indicated that Ce/Gd@HA/PLGA composites significantly promoted the performance of MC3T3-E1 cells, containing proliferation, adhesion, and osteogenic differentiation capacities. These include the improvement of alkaline phosphatase activity, enhancement of mineral deposition, and upregulation of OCN and COL-1 gene expression. The *in vivo* experimental results demonstrated that the Ce/Gd@HA/PLGA composites significantly improved the healing rate of rat bone defects. The MRI images indicated that the Ga-doped composites were observed in the MRI T1 sequence in rats. The aforementioned results suggested that Ce/Gd@HA/PLGA composites not only effectively promoted bone formation but also enhanced MRI capability. The composites synthesized in this study have great potential in bone regeneration with an extensive application in bone tissue engineering.

## Introduction

Hydroxyapatite is a major inorganic component in human and animal bones (Palmer et al., 2008; Yang et al., 2020). As an important implant and scaffolding material, it has substantial clinical potential in medicine, dentistry, and orthopedics. A large number of studies have shown that synthetic HA has certain solubility in the body and can release harmless ions and stimulate or induce bone tissue regeneration, showing good biocompatibility (Huang et al., 2015). Among nanomaterials, synthetic HA nanoparticles (NPs) have a low volume, large specific area, and improved biodegradability and bioactivity (Pandi and Viswanathan, 2015; Roh et al., 2015).

However, hydroxyapatite has its own shortcomings, such as poor mechanical properties and low osteogenic induction capability. At present, X-ray and CT are often used to observe HA materials, but the density of HA is similar to bone density, resulting in difficulty to distinguish them in the images. During this time, X-ray and CT will also cause uncertain radioactive damage to the human body (Costello et al., 2013). The exceptional advantage of MRI lies in the absence of radiological injury to the human body and its good imaging ability of the bone structure in both qualitative and quantitative aspects (Nishimura et al., 2021; Soldati et al., 2021). In addition to HA, the inorganic contents of human bone tissues also contain a variety of trace elements, including Ce, zinc, magnesium, silicon, strontium, and sodium ions. Therefore, it is of great significance in the field of bone repair and tissue engineering to synthesize HA compounds with target ions to construct a new material with both better osteogenic induction and MRI ability.

Rare earth elements have been widely used in the fields of microelectronics, machinery, energy, and biomedicine due to their excellent chemical and physical properties (Aswathy et al., 2015; Montini et al., 2016). Cerium is one of the rare earth elements with low cytotoxicity, osteogenic induction capability, and anti-inflammatory (Gudasi et al., 2007) and antitumor abilities (Fricker, 2006). Its excellent biomedical properties have been verified and applied in various clinical studies. The metabolism of bone cells can be accelerated by only a spot of cerium ions (0.001 μM) (Priyadarshini et al., 2017). It has been demonstrated that Ce^3+^ has high biosafety (Xiang et al., 2019). Doping the synthesized nanomaterials with Ce^3+^ can effectively induce differentiation of mesenchymal stem cells (MSCs) into osteoblasts, subsequently promoting the formation of new bones (Naganuma and Traversa, 2014). The ionic radius of Ce^3+^ (1.034 Å) is very close to that of Ca^2+^ (0.99 Å). This similarity enables Ce^3+^ to replace Ca^2+^ in many calcium-containing compounds (Celardo et al., 2011).

Meanwhile, the rare earth element gadolinium has an attractive paramagnetic effect caused by its seven unpaired 4f electrons, so Gd^3+^ and its compounds can be used as MRI contrast agents to enhance the contrast of the images (Zhang et al., 2009; Zhu et al., 2016). A number of studies have shown that incorporating Gd^3+^ may enhance MRI capacity of composites, such as Gd NP–diethylenetriaminepentaacetic acid (Gd NPs-DTPA)-terminated dendrimer (Langereis et al., 2004) and Gd ions entrapped in zeolites (Platas-Iglesias et al., 2002). It has been demonstrated that calcium phosphate tribasic materials doped with Gd^3+^ exhibited ferromagnetic properties and then contributed to excellent MRI performance (Shen et al., 2015; Yang et al., 2020).

There are two crystallographically distinct Ca loci in HA unit cells: Ca (I) and Ca (II). During the synthetic process, those Ca^2+^ ions could be replaced by other cations. It is reported that both Ca (I) and Ca (II) can be replaced by rare earth element ions and alkali earth metal ions, such as Mg^2+^, Fe^2+^, Sr^2+^, Ba^2+^, Na^+^, K^+^, and Ag^+^ cations, which lead to new performance of HA (Vukomanović et al., 2011; Fihri et al., 2017; Malakooti et al., 2018; Wang et al., 2020; Liu et al., 2021). Until now, to the best of our knowledge, there were no reports on hydrothermal synthesis of Ce^3+^ and Gd^3+^ co-doped HA NPs.

In this study, we successfully synthesized Ce/Gd co-doped HA NPs with osteogenic induction and MRI abilities by using the hydrothermal synthesis method. The influence of doping Ce^3+^ and Gd^3+^ on the HA nanostructures and MRI ability was investigated. Ce/Gd@HA NPs and PLGA were used to synthesize Ce/Gd@HA/PLGA composite scaffolds with biocompatibility and biodegradability. The cytotoxicity, biocompatibility, osteogenic induction ability, and MRI ability of the synthesized scaffolds were also studied in the present work.

## Experimental Details

### Materials

PLGA (LA/GA = 80:20, Mn = 1 × 10^5^) was synthesized in our laboratory by the ring-opening copolymerization (ROP) of L-lactide (LA) and glycolide (GA), which were purchased from Purac (the Netherlands). N-methyl-pyrrolidone (NMP), cerium nitrate hexahydrate (Ce(NO_3_)_3_·6H_2_O, 99%), and gadolinium nitrate hexahydrate (Gd(NO_3_)_3_·6H_2_O, 99%) were purchased from Aladdin Biochemical Technology Co., Ltd. (Shanghai, China). Chloroform was purchased from Beijing Chemical Works (Beijing, China).

### Preparation of Ce/Gd Co-Doped Hydroxyapatite

Ca loci in HA unit cells were replaced by Ce and Gd ions, and then HA composites were synthesized by using the hydrothermal method. This reaction process is described by the following formula:

$$\left(\frac{20-3x-3y}{2}\right)Ca^{2+}+xCe^{3+}+yGd^{3+}+6PO_{4}^{3-}+2OH^{-}=Ca_{\left(\frac{20-3x-3y}{2}\right)}Ce_{x}Gd_{y}{\left(PO_{4}\right)}_{6}{\left(OH\right)}_{2}.$$

Here, seven samples were synthesized by using the hydrothermal method. They are HA, 0.5Ce@HA, 1Ce@HA, 0.5Gd@HA, 1Gd@HA, 0.5Ce/1Gd@HA, and 1Ce/1Gd@HA. Solid raw materials containing calcium nitrate, gadolinium nitrate hexahydrate, cerium nitrate hexahydrate, and ammonium dihydrogen phosphate were dissolved in deionized water at a concentration of 1 mol/L. The aforementioned four aqueous solutions were poured into the reaction kettle, according to the feeding molar ratios in Table 2. The volume of the reaction kettle is 100 ml, and the amount of solution for each reaction is 50 ml. Ammonia water was used to maintain the pH of the hydrothermal solution within 10.0–11.0. The mixture was magnetically stirred for 1 h in a water bath with a temperature of 40°C until the white suspension was generated. Then the suspension with a volume of 50 ml was reacted in a sealed autoclave at a temperature of 180°C for 8 h. After cooling down to room temperature, the reactant was centrifuged, and the supernatant was removed and sonicated (3 times). The final solution was obtained after drying under a temperature of −40°C in a vacuum freeze dryer for 48 h.

### Configuration of the Ce/Gd@HA/PLGA Composites

Nanocomposite membranes containing Ce/Gd@HA/PLGA were prepared by using a solvent mixing method using N-methyl-pyrrolidone (NMP) as a solvent. Specifically, the Ce/Gd@HA powder was added to NMP and dissolved with the help of a magnetic mixer. PLGA was added to the suspension and fully dissolved by magnetic stirring in a water bath. The suspension was then poured onto a round, clean glass substrate to form a thin film and dried at a temperature of 50°C for 10 h.

For better MRI observation, nanocomposite scaffolds containing 20 wt% Ce/Gd@HA/PLGA were fabricated by using the solvent mixing method. First, Ce/Gd@HA composites were dispersed into NMP by magnetic stirring and ultrasonic vibration. The mass ratio of NMP to Ce/Gd@HA/PLGA was 4:1. The suspension was then extracted with a 2.5-ml syringe and frozen at a temperature of −80°C for 12 h. The frozen scaffolds were placed into purified water with a volume of 500 ml for replacement. Purified water was changed every 8 h for 3 consecutive days. After the replacement, the scaffolds were dried at a temperature of −40°C for 12 h, and finally, Ce/Gd@HA/PLGA scaffolds were fabricated.

### Characterization of the Ce/Gd@HA NPs

The molar ratio of Ce^3+^ to Gd^3+^ and Ca^2+^ in doped nanocrystals was measured by inductively coupled plasma (ICP) atomic emission spectroscopy. XRD data ranging from 20 to 55° (2θ) were obtained to analyze the crystal structures of Ce/Gd@HA NPs using a D8 Advance diffractometer (Bruker Co., Ltd., Germany). FTIR spectroscopy was performed using a Bio-Rad Win-IR spectrophotometer (Watford Co., Ltd., United Kingdom), and the chemical components of Ce/Gd@HA NPs were investigated using potassium bromide (KBr) microtomy. The microstructures of Ce/Gd@HA NPs and their sizes were observed and measured using an environmental scanning electron microscope (ESEM, XL30 FEG, Philips). The size distribution of the NPs was calculated using the ImageJ (National Institutes of Health, United States) based on the ESEM images. The magnetic properties of Gd@HA NPs at 293 K were measured using a vibrating sample magnetometer (MMPS-XL-7, Quantum Design, China).

### Characterization of the Ce/Gd@HA/PLGA Scaffolds

ESEM (XL30 FEG, Philips, the Netherlands) was used to observe and measure the microstructure of the scaffold. Micro-computed tomography was carried out to analyze the composites by micro-CT (Bruker, SkyScan1172, Germany). Based on micro-CT images, the porosity of NPs was measured using CTAn software. T1-weighted MRI images of the composite were obtained using a 3.0T scanner (Siemens Magnetom Avanto, Germany).

### Cell Culture

Fetal bovine serum (FBS) (10%, Gibco, United States), 10 mm HEPES (Sigma, United States), 100 mg/L streptomycin (Sigma, United States), and 63 mg/L penicillin (Sigma) were added to Dulbecco’s minimum basic medium (Hyclone, United States). Mouse pre-osteoblast MC3T3-E1 cells (Shanghai Institutes for Biological Sciences, Chinese Academy of Sciences) were kept on the nanocomposite matrix in a humid atmosphere at 37°C and 5% CO_2_.

### In Vivo Rat Study

In this study, 8-week-old male Sprague Dawley rats were used to analyze the level of bone repair 4–8 weeks after implantation. All rats were kept in animal facilities for at least 1 week to acclimatize before *in vivo* experiments. The rats were euthanized at 4^th^ and 8^th^ weeks after surgery. Animal experiments were carried out following the NIH Guidelines for the Care and Use of Laboratory Animals, provided by Jilin University, Changchun. We guarantee that this study was conducted in accordance with international, national, and guidelines and also animal experiments, clinical research, and biodiversity rights. The protocol in this study was approved by the Department of Orthopedics, China-Japan Joint Hospital, Jilin University, Changchun, China.

### Surgical Procedures

In total, 24 rats with 250–300 g each were provided by Jilin University. They were evenly divided into four groups: HA/PLGA, 1Ce@HA/PLGA, 1Gd@HA/PLGA, and 1Ce/1Gd@HA/PLGA groups. All rats were treated with ultraviolet radiation for 6 h before surgical experiments. The anesthetized rats were supine and fixed on the surgical corkboard. Then the hind limbs of the rats were shaved and disinfected with iodine. The skin near the tibial bone was cut longitudinally to separate the subcutaneous tissues from the tibia. A circular hole defect was created in the tibia by a hand drill. HA/PLGA, 1Ce@HA/PLGA, 1Gd@HA/PLGA, and 1Ce/1Gd@HA/PLGA scaffolds were implanted with a diameter of 2.5 mm and a depth of 6 mm. The rats were then euthanized at 4^th^ and 8^th^ weeks after surgery. After wound suturing, the rats were subcutaneously injected with 300,000 units/day of penicillin within 3 days.

Bone repair of the tibial defect was evaluated using microcomputed tomography equipment at 4^th^ and 8^th^ postoperative weeks. Tibial CT scan images of the rats in the four groups were reconstructed using a 3D viewer, and the volume changes in the new bone near the implanted scaffolds were observed. The images were reconstructed for quantitative analysis of regions of interest using CTvox software (Bruker, Skyscan, Germany). The bone volume/tissue volume (BV/TV) was measured by CTAn software. After micro-CT treatment, sections of the tibia new bone, heart, liver, spleen, lung, kidney, and brain were stained with H&E staining, and Masson trichromatic staining was used for histological analysis.

### Statistical Analysis

Three repeated measurements were taken, unless otherwise stated. The obtained data were expressed as mean value ± standard deviation. Origin software was used for one-way analysis of variance to get the statistical results. A *p*-value marked with as asterisk (*) less than 0.05 indicates significant difference.

## Results and Discussion

### Characterization of the Nanoparticles

#### Physicochemical Characterization

**FIGURE 1:**
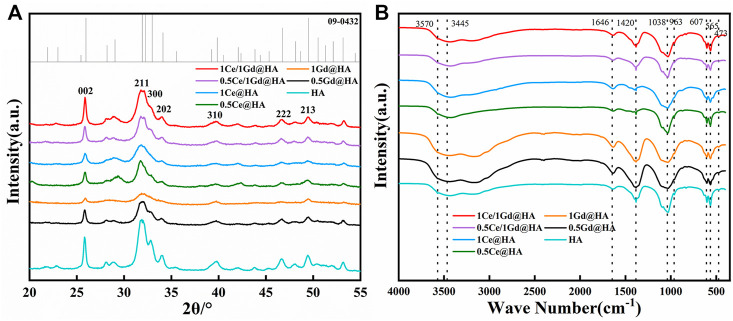
**(A)** FTIR spectra and **(B)** XRD step scan patterns from 20° to 55°.

A variety of groups existed on the surface of HA, such as the hydroxyl group and the phosphate group. Figure 1A shows the FTIR images for HA doped with Ce^3+^ and Gd^3+^. The bands at 963 and 1,038 cm^−1^ correspond to the P-O telescopic vibration and bending vibration. The band at 473 cm^−1^ is produced by the O-P-O bending vibration. The bands at 565 and 607 cm^−1^ are due to the O-P-P bending vibration (Zhu et al., 2016). The wide bands at 3,570 and 3,445 cm^−1^ and the narrow band at 1,646 cm^−1^ belong to the O–H telescopic vibration and bending vibration, indicating the presence of -OH in the sample.

The location of the aforementioned functional groups indicates that the main groups in the samples are hydroxy and phosphate groups. The characteristic peaks of HA from all the samples are shown.

By comparing the crystal morphology of HA (PDF: 09-0432), all components present the characteristic XRD peaks. From Figure 1B, the diffraction patterns of all doped samples are similar to those of HA, and there are no diffraction peaks of other impurities, indicating that the samples are pure. It demonstrates that the crystal structure of HA doped with Ce^3+^ and Gd^3+^ is almost the same as that of native HA. Except for the 1Gd@HA sample, all components have high crystallinity. For the single doped samples (Ce^3+^ or Gd^3+^), shown as blue, green, yellow, and black lines, the diffraction peaks become blunt at the location marked in the figure (213, 222, 300, and 310). This indicates that the high doping concentration correlates to the significant effect on the crystal structure of HA. More narrow peaks occur in the curve of 1Ce@HA than that of 1Gd@HA. This indicates that Gd affects the crystal structure of HA more significantly than Ce. The ionic radius of Ce^3+^, Ca^2+^, and Gd^3+^ is 1.034 Å, 1.00 Å, and 0.938 Å, respectively. The difference in the ionic radius between Gd^3+^ and Ca^2+^ is 0.07 Å, while the difference in the ionic radius between Ce^3+^ and Ca^2+^ is 0.03 Å. Thus, HA crystal structures are more significantly affected by doping of Gd^3+^. The doping of Gd^3+^ with a small ionic radius leads to a decrease in the particle size. Obvious narrower peaks occur for both 0.5Ce/1Gd@HA and 1Ce/1Gd@HA samples than those for single doped samples. Combined with the ionic radii and the related XRD results, it can be deduced that the HA crystal could be less affected by co-doping with Ce^3+^ and Gd^3+^ during the synthesis process. Ce^3+^ has a large ionic radius, while Gd^3+^ has a small one (Ce^3+^ > Ca^2+^ > Ga^3+^). The co-doping of them makes the change of the HA crystal structure less altered. Liu et al. (2021) also reported a similar phenomenon that the doping of Mg^2+^ with a larger ionic radius balanced the effect of incorporation of Ba^2+^ with a smaller ionic radius on the HA crystal structure.

The lattice parameters and cell volume of HA will change as Ca^2+^ is replaced by Ce^3+^ and Gd^3+^. a-Axis, b-Axis, c-Axis, and unit cell volume of the synthesized nanoparticles were calculated using the Scherrer formula and Jade 6.5 software. The calculated results are shown in Table 1 (a-Axis = b-Axis). Only in Gd-doped component, the crystal size on a-Axis and c-Axis, a/c value, and cell volume gradually decreased with the increase in the Gd doping amount. The a/c ratio of the lattice is reduced by a decrease in the a-Axis, thus affecting the hexagonal structure of the HA crystal. In addition, the volume reduction range of Ce/Gd co-doped HA was significantly lower than that of the Gd-doped sample, indicating that the doping of Ce balanced the effect of Gd doping on the HA crystal structure. The calculated results agreed with XRD measurement results.

**TABLE 1:** Crystallographic parameters of Ce/Gd co-doped HA.

Sample	a (Å)	c (Å)	a/c	Vol. (Å^3^)
HA	9.4422	6.8926	1.3699	532.19
0.5Gd@HA	9.4184	6.8912	1.3638	530.10
1Gd@HA	9.3510	6.8879	1.3576	529.54
0.5Ce@HA	9.4493	6.8946	1.3705	532.50
1Ce@HA	9.4664	6.9047	1.3710	532.66
0.5Ce/1Gd@HA	9.4284	6.8921	1.3680	530.60
1Ce/1Gd@HA	9.4300	6.8926	1.3681	531.82

Ce/Gd co-doped HA could be indicated using a simple chemical formula: Ca_(10-1.5x-1.5y)_Ce_x_Gd_y_(PO_4_)_6_(OH)_2_, where x and y represent the replacing percentage of Ca ions by Ce and Gd ions. For all samples, the original feed-to-mole ratio (Ca:Ce:Gd) was not equal to the final ratio after synthesis (Yuan et al., 2013). The final Ca^2+^, Ce^3+^, and Gd^3+^ ratios in the synthesized composites are shown in Table 2. The final doping ratios are unequal to the theoretical ones. This phenomenon may be caused by the radius differences among Ca, Ce, and Gd ions. As doping concentration changes, the volume of HA unit cells increases or decreases, eventually changing the deformity degree of the crystal cells (Fernandez et al., 2017). Therefore, in the present work, the doping concentration of Ce^3+^ and Gd^3+^ is higher than that of the original feeding ratio, while for Ca^2+^, it is lower than that of the original one. This also indicates that replacing Ce^3+^ and Gd^3+^ with HA crystal cells is more dominant than Ca^2+^ during the ion exchange process (Eriksson et al., 2018). The Gd doping concentration for 1Gd@HA composites is higher than the Ce doping concentration for 1Ce@HA, which is consistent with the XRD results. The comparison between 1Ce/1Gd@HA and 1Gd@HA groups demonstrates that the Gd^3+^ content drops with the increase in the Ce doping concentration. Thus, HA crystal structures are less affected due to lower Gd^3+^ content.

**TABLE 2:** Chemical component analysis of Ca, Ce, and Gd in Ce/Gd@HA and HA NPs.

Sample	Feeding molar ratios	Ca/P in theory	(Ca + Ce + Gd)/P in theory	Chemical formula in theory	Molar ratios in reality	Chemical formula in reality by ICP
HA	Ca/Ce/Gd				Ca/Ce/Gd	
	10/0/0	1.67	1.67	Ca_10_(PO_4_)_6_(OH)_2_	10/0/0	Ca_10_(PO_4_)_6_(OH)_2_
0.5Gd@HA	9.25/0/0.5	1.54	1.67	Ca_9.25_Gd_0.5_(PO_4_)_6_(OH)_2_	10/0/2.09	Ca_7.60_Gd_1.59_(PO_4_)_6_(OH)_2_
1Gd@HA	8.5/0/1	1.42	1.67	Ca_8.5_Gd_1_(PO_4_)_6_(OH)_2_	10/0/9.26	Ca_4.19_Gd_3.88_(PO_4_)_6_(OH)_2_
0.5Ce@HA	9.25/0.5/0	1.54	1.67	Ca_9.25_Ce_0.5_(PO_4_)_6_(OH)_2_	10/1.61/0	Ca_8.05_ Ce _1.3_(PO_4_)_6_(OH)_2_
1Ce@HA	8.5/1/0	1.42	1.67	Ca_8.5_Ce_1_(PO_4_)_6_(OH)_2_	10/3.2/0	Ca_6.75_ Ce _2.16_(PO_4_)_6_(OH)_2_
0.5Ce/1Gd@HA	7.75/0.5/1	1.29	1.67	Ca_7.75_Ce_0.5_Gd_1_(PO_4_)_6_(OH)_2_	10/1.29/3.79	Ca_5.68_Ce_0.73_Gd_2.15_(PO_4_)_6_(OH)_2_
1Ce/1Gd@HA	7/1/1	1.16	1.67	Ca_7_Gd_1_Ce_1_(PO_4_)_6_(OH)_2_	10/3.01/4.49	Ca_4.79_Ce_1.44_Gd_2.03_(PO_4_)_6_(OH)_2_

**FIGURE 2:**
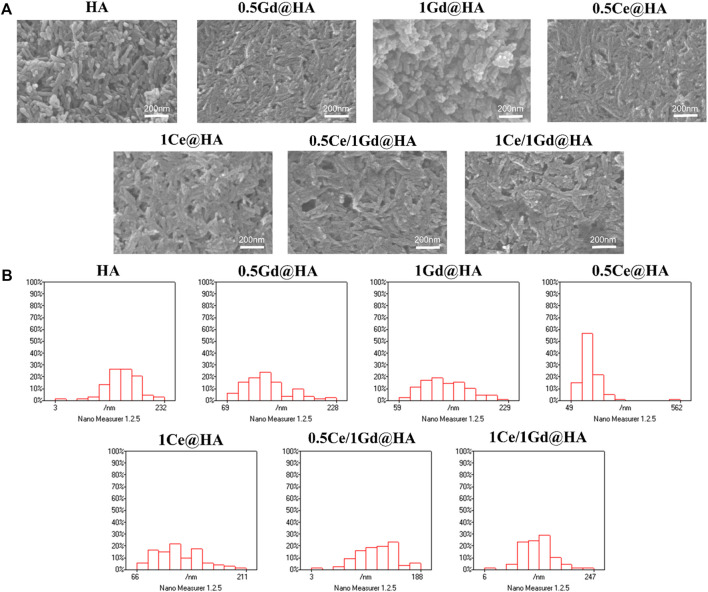
**(A)** SEM images and **(B)** diameter distribution histograms of HA, 0.5Ce@HA, 1Ce@HA, 0.5Gd@HA, 1Gd@HA, 0.5Ce/1Gd@HA, and 1Ce/1Gd@HA nanoparticles.

To further analyze the morphology of the synthesized composites, ESEM analysis was performed. Figure 2A shows the ESEM images of the synthesized composites with different doping concentrations. The crystal structure of HA has a short rod-like structure. Interestingly, the crystal structure of 1Gd@HA composites changed, exhibiting an unevenly stacked particle shape with rounded ends. It can be seen that the contrast of the lattice becomes less apparent as the Gd content increases, which may indicate partial damage to the lattice structure. This conclusion is similar to that obtained from XRD images. The XRD diffraction peak for the 1Gd@HA group is much lower and smoother than that for other groups, indicating the decrease in HA crystallinity. However, homogeneous rod-like structures could be observed again in the Ce/Gd co-doped groups. Thus, the introduction of Ce prevents the HA crystal from damage after Gd doping. For 0.5Ce@HA and 1Ce@HA composites, homogeneous rod-like structures could still be observed in ESEM images. This demonstrates that the doping of Ce could hardly affect the original structure of the HA crystal.

To further verify the change in the particle size of HA NPs, Nano Measure software was used to perform statistical analyses on ESEM images. Figure 2B shows that the size of nanoparticles decreases with doping of Gd. The size of Ce/Gd@HA composites was larger than that of the same feeding Gd@HA composites. This indicates that Ce doping reduces the effect of Gd on the size change of HA NPs, which is consistent with the XRD results (Eriksson et al., 2018).

The ESEM analysis results indicate that doping of Gd^3+^ with high concentration leads to the change of HA lattice. After doping of both Ce^3+^ and Gd^3+^ simultaneously, the HA crystal structure was not destroyed, maintaining its original crystal structure.

**FIGURE 3:**
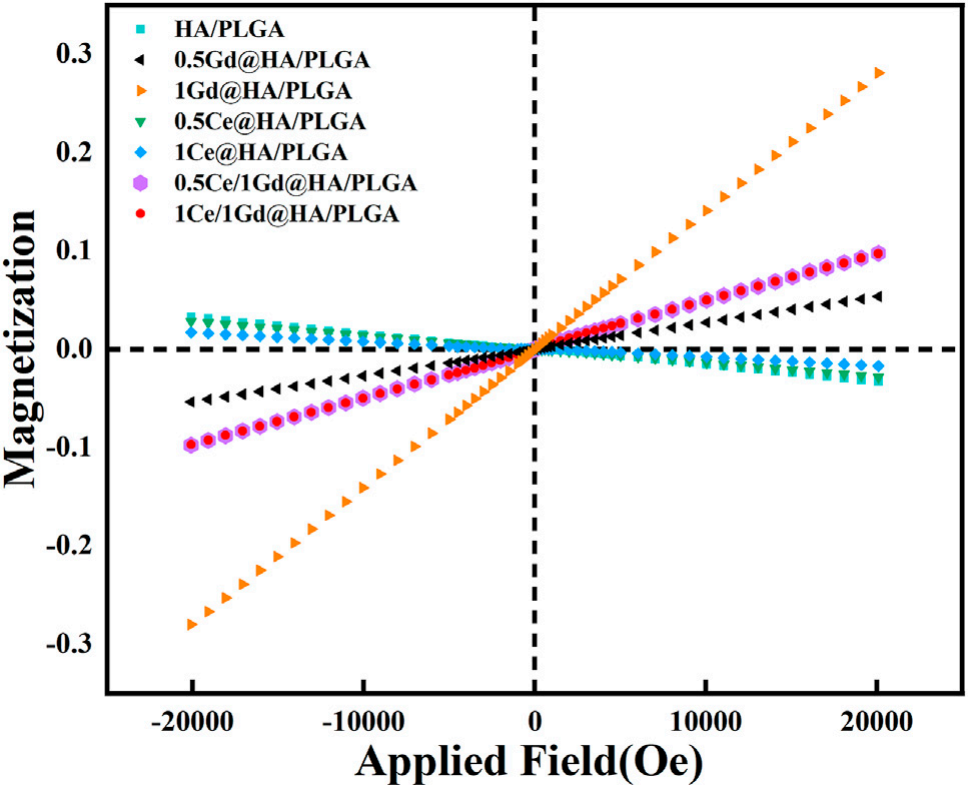
Magnetization curves for synthesized HA, 0.5Ce@HA, 1Ce@HA, 0.5Gd@HA, 1Gd@HA, 0.5Ce/1Gd@HA, and 1Ce/1Gd@HA NPs.

In this work, the goal of doping of Gd^3+^ into the composites is to produce advanced nanocomposites that can be detected by MRI. Thus, it is critical to measure the magnetism of the synthesized NPs. As shown in Figure 3, all the magnetization curves for the 0.5Gd@HA, 1Gd@HA, 0.5Ce/1Gd@HA, and 1Ce/1Gd@HA NPs are linear, indicating that all aforementioned samples are paramagnetic. The magnetism of the NPs increased with the improvement of Gd^3+^ doping concentration. Since Ce is free of magnetism and Ce/Gd@HA NPs do show magnetism, it indicates that Gd^3+^ was successfully doped into the HA lattice. The magnetization curve of the 0.5Ce/1Gd@HA sample is the same as that of the 1Ce/1Gd@HA sample. It indicates the equal Gd doping amount in those two samples, which agrees with the result obtained by ICP tests. HA, 0.5Ce@HA, and 1Ce@HA NPs exhibit diamagnetic ability. In general, the NPs exhibit excellent paramagnetism when doping Gd^3+^ to HA by using the hydrothermal method (Miao et al., 2018).

**FIGURE 4:**
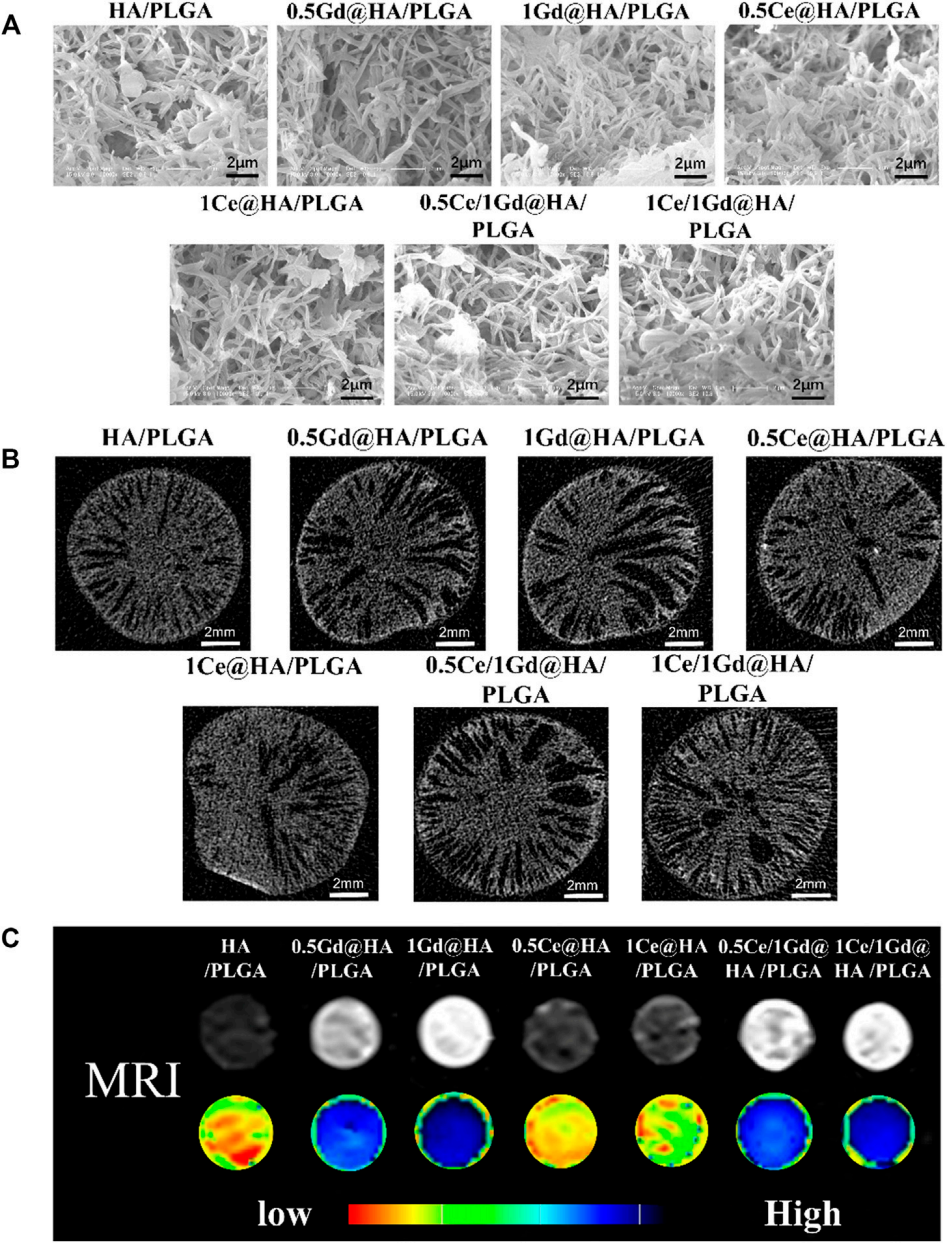
**(A)** ESEM micrographs of porous scaffolds. **(B)** Micro-CT axial scanning images, **(C)** T 1 weighted images, and parametric mapping of HA/PLGA, 0.5Ce@HA/PLGA, 1Ce@HA/PLGA, 0.5Gd@HA/PLGA, 1Gd@HA/PLGA, 0.5Ce/1Gd@HA/PLGA, and 1Ce/1Gd@HA/PLGA.

As shown in Figure 4A; Supplementary Figure S1 (ESI), the nanocomposite scaffold is a porous structure with an aperture mainly distributed at 0.5 μm. The aperture sizes and the number of scaffolds are similar in all samples. It is reported that the structure of the scaffolds could affect both their mechanical properties and the improvement of new bone formation. The formation of micropores is probably affected by the leaching of the NMP solution. The formation of Ce/Gd@HA/PLGA composites was hardly affected by the doping of Ce^3+^ and Gd^3+^, since the morphology of the composites is the same as that of HA/PLGA composites.

Axial scanning images of the scaffolds captured by micro-CT show that the connected apertures extend from the center to outside, that is, typical “finger” pore structures occur after scaffold fabrication by using the phase separation method, as shown in Figure 4B. The nanoparticles disperse evenly on the scaffolds without agglomerated particles. The loose porous structures are evenly distributed on the fracture surface of the scaffolds, providing an excellent growing environment for bone tissues.

CTAn software was used to analyze the porosity of the scaffolds. From Supplementary Figure S2 (ESI), it can be found that the porosities of most scaffolds are about 50 % without statistical differences among the groups. Endophytic cells could grow inside the pore structure of the scaffolds, while the interconnected channel inside the scaffolds could allow the humoral fluids and blood vessels to grow into the scaffolds. It contributes to the sufficient exchange of oxygen and nutrients and is also beneficial to repair the tissue around the defect (Chen et al., 2015).

While the composite material can be clearly observed by MRI after implantation, it will contribute to a broader range of applications. In this study, Ga^3+^ doping provided the material with advanced tracer properties, which are essential for MRI applications. Since the Gd-doped material has enhanced paramagnetism (Figure 3), synthesized with PLGA, it could be used for MRI. To evaluate the imaging ability of the nanocomposites, MRI tests were performed using those scaffolds immersed in deionized water, as shown in Figure 4C. As predicted, the composites doped with Gd^3+^ could be clearly observed in MRI images according to T1-weighted images and parametric mapping (Sigg et al., 2016). The signal intensity of the T1-weighted images for Gd^3+^-doped composites is much higher than the others (HA/PLGA, 0.5Ce@HA/PLGA, and 1Ce@HA/PLGA). The signal intensity is highest for the 1Gd@HA/PLGA group due to the high doping concentration of Gd elements. These results indicate that the doping of Gd^3+^ may improve the MRI capability of the composites, which is dependent on its doping dose. However, the signal intensities between HA/PLGA and Ce@HA/PLGA groups are almost the same, indicating that doping of Ce^3+^ cannot enhance MRI images. The aforementioned results demonstrate that the Ce/Gd@HA/PLGA composites could be used for commercial MRI.

### Biological Evaluation of the Ce/Gd@HA/PLGA Nanocomposites

#### Evaluation of Nanoparticle Biocompatibility

**FIGURE 5:**
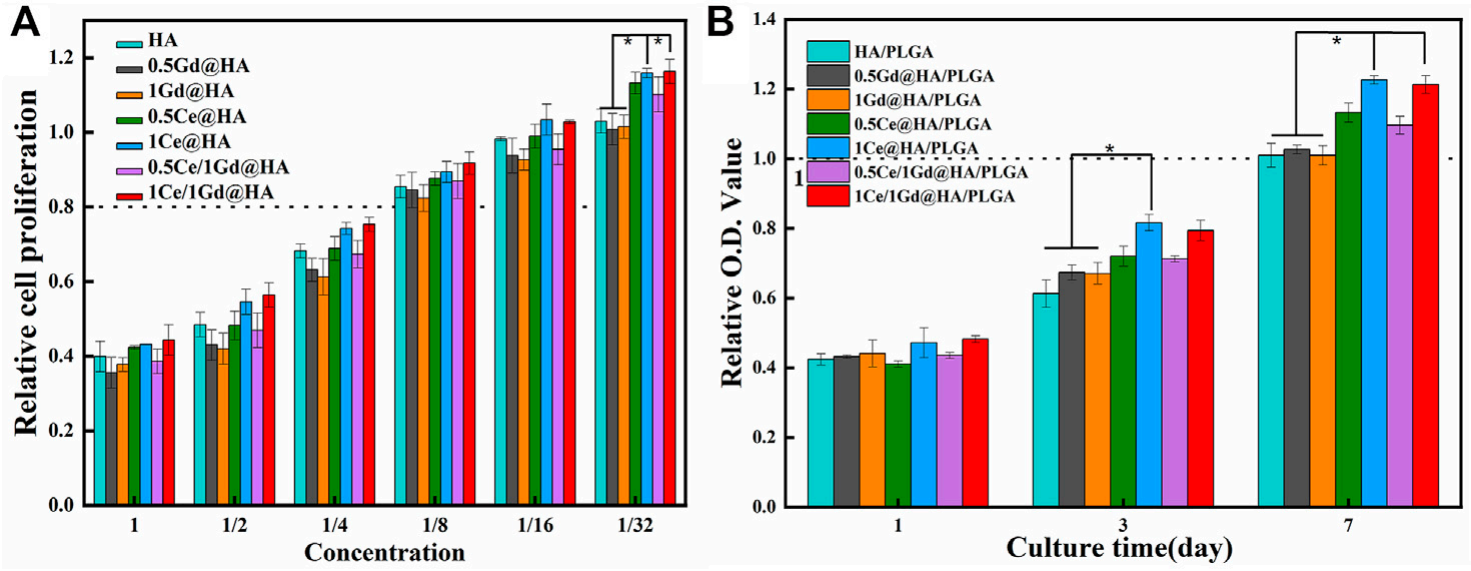
**(A)** Proliferation of MC3T3-E1 cells cultured for 24 h in each solution of HA, 0.5Ce@HA, 1Ce@HA, 0.5Gd@HA, 1Gd@HA, 0.5Ce/1Gd@HA, and 1Ce/1Gd@HA nanoparticles with different concentrations. **(B)** CCK-8 assays of MC3T3-E1 osteoblasts on 1, 3, and 7 days by using different nanocomposites. * *p* < 0.05.

HA is an FDA-certified biological material with excellent biocompatibility. However, Gd, like heavy metals, might be toxic to cells and human bodies (Sharma et al., 2006). Some Gd-doped composites with high doping concentration exhibit adverse biological effects (Todd and Kay, 2008). In an attempt to evaluate the effect of Ce/Gd@HA NPs on biocompatibility, those nanoparticles were used to culture MC3T3-E1 cells by using the CCK-8 method for evaluating the vitality of the cells. HA nanoparticles were immersed in the sterile DMEM at a ratio of 20 mg/ml and incubated in an oscillating box at 37°C for 24 h. After centrifugation at 120 rpm, the supernatant was extracted as the extraction solution. MC3T3-E1 cells were cultured in a fresh DMEM for 24 h, and then the DMEM was discarded. The extract stock solution and gradient dilution solution (1/2, 1/4, 1/8, 1/16, and 1/32) were added for further culture for 24 h In total, 100 μL of the prepared CCK-8 reagent was added to each well after 24 h and then incubated for 2 h at 37°C (avoiding light). Then the absorbance of the mixed solution at wavelength of 450 nm was measured using a multifunction microplate tester. As shown in Figure 5A, 40–50 % of cells survived in undiluted solution for all groups. As a solution diluted 8 times, over 80 % of cells survived, indicating the obvious toxicity decrease in the NPs. When the solution was diluted 32 times, all cells proliferated well. Cell viability of the group for Ce^3+^-doped NPs is much higher than that without Ce^3+^ doping (*p <* 0.05). Therefore, the leaching solution exhibited slight toxicity after diluting 8 times for HA and Ce/Gd@HA NPs. The cell viability could be improved by doping of Ce^3+^.

The low toxicity may be due to the high crystallinity of the synthesized HA NPs, which results in excellent Gd^3+^ adherence to the surface of the synthesized NPs and then the heavy metal ion will hardly dissolve in the surrounding water. This is consistent with previous reports that NPs with high crystallinity have good chemical stability and low toxicity (Kuang et al., 2014; Huang et al., 2016; Zhu et al., 2016). The concentration of NPs was decreased, since during Ce/Gd@HA/PLGA preparation, the mass ratio for NPS and PLGA was 1:4. In addition, NPs are partially clad by PLGA. At the initial stage, only the NPs on the scaffold surface could contact the cells and then slowly release as the scaffold degraded. Thus, the toxicity of NPs is significantly reduced to a negligible value.

### Evaluation of Cell Proliferation

Ideal nanocomposite scaffolds could be used in clinical applications with high biological viability, such as excellent cell attachment, cell diffusion, and cell proliferation capacities. To evaluate the cell proliferation in nanocomposites, the viabilities of MC3T3-E1 cells growing on varying culture mediums were measured. As shown in Figure 5B, the optical density (OD) increases for all groups with increased culture duration, indicating that the cells could proliferate in all nanocomposites. At a cultural duration of 1 day, there was no significant difference among all groups. After 3 days, the OD values for 1Ce@HA/PLGA groups were much higher than those for HA/PLGA, 0.5Gd@HA/PLGA, and 1Gd@HA/PLGA groups (*p* < 0.05). This may be related to the highest Ce content in 1Ce@HA/PLGA (Chou et al., 2005). After 7 days, all components had a higher rate of cell proliferation than the blank control group (100%). For 1Ce@HA/PLGA and 1Ce/1Gd@HA/PLGA groups, the OD values are much higher. Schmidlin et al. (2012) reported that low-level Ce^3+^ doping may also promote proliferation and differentiation of MC3T3-E1 cells *in vitro*. Thus, the newly synthesized HA/PLGA scaffolds in the present study cannot repress the cell proliferation. After doping with Ce, the cell proliferation could be further improved. Cell proliferation for the Gd-doped group is almost the same as that for the HA group. Agreeing with the aforementioned toxicity test results, the doping of Gd did not lead to cytotoxicity, although Gd is widely regarded as a cytotoxic element.

**FIGURE 6:**
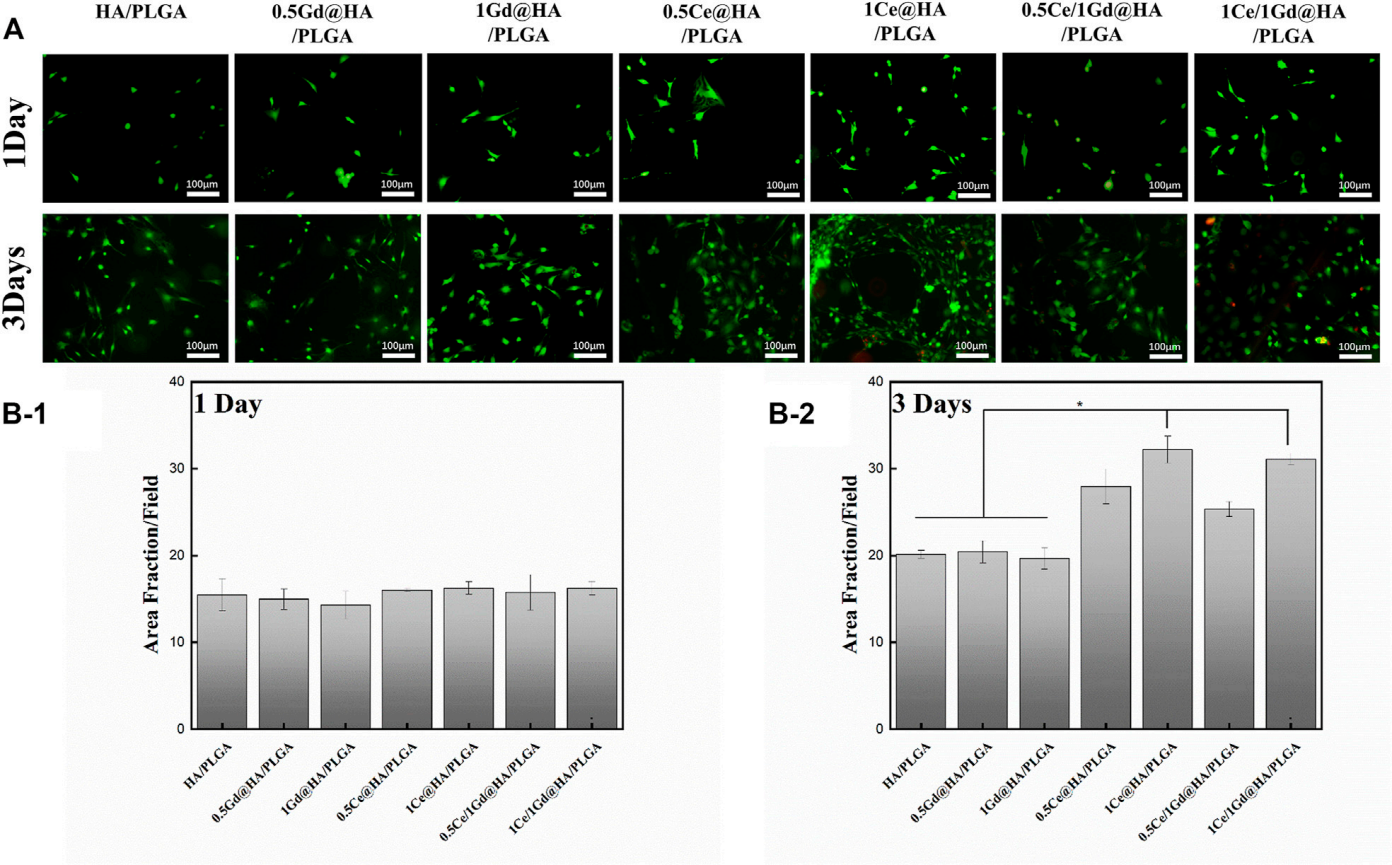
**(A)** Comparison of morphology of MC3T3-E1 cells after 1 and 3 days, which were cultured on HA/PLGA, 0.5Ce@HA/PLGA, 1Ce@HA/PLGA, 0.5Gd@HA/PLGA, 1Gd@HA/PLGA, 0.5Ce/1Gd@HA/PLGA, and 1Ce/1Gd@HA/PLGA mediums. **(B-1 and B-2)** Average fluorescence intensity of MC3T3-E1 cells cultured on varying mediums on 1 and 3 days.* *p* < 0.05.

The results of the calcein/PI fluorescence test are shown in Figure 6A, and the results were similar to the proliferation results. As shown in Figure 6B–1, there was no significant difference in the fluorescence staining intensity between the groups on the first day (*p* > 0.05). Figures 6B–2 shows the fluorescence intensity of 1Ce@HA/PLGA on the third day, which is higher than HA/PLGA, 0.5Gd@HA/PLGA, and 1Gd@HA/PLGA groups (*p* < 0.05), showing a concentration-dependent effect. There is no difference between 1Ce@HA/PLGA and 1Ce1Gd@HA/PLGA (*p* < 0.05). It is shown that the fluorescence intensity of the Gd@HA/PLGA sample is closer to that of the HA/PLGA sample (Hu et al., 2014).

### Cell Adhesion

**FIGURE 7:**
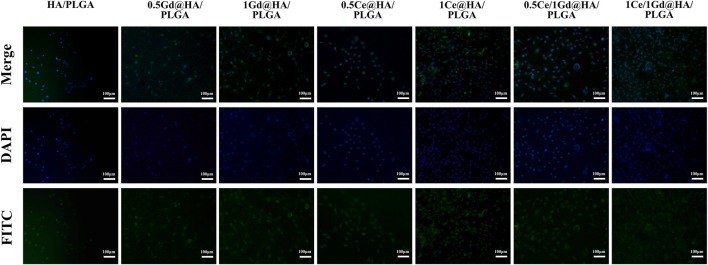
Fluorescence micrographs of MC3T3-E1 cells on HA/PLGA, 0.5Ce@HA/PLGA, 1Ce@HA/PLGA, 0.5Gd@HA/PLGA, 1Gd@HA/PLGA, 0.5Ce/1Gd@HA/PLGA, and 1Ce/1Gd@HA/PLGA nanocomposites cultured for 24 h. Cells were stained with FITC (green) and DAPI (blue). Scale bar, 100 μm.

Cell adhesion is an important indicator for the biocompatibility of reaction materials and critical for the cell proliferation and differentiation applications. Fluorescein isothiocyanate (FITC) is a commonly used fluorescent dye, which can combine with specific proteins and exhibit bright yellow–green fluorescence. After the MC3T3-E1 cells were co-incubated with the synthesized material for 24 h, the adherence of the cells to the surface of the material was evaluated by FITC staining. Figure 7 shows the adhesion of MC3T3-E1 cells to the material surface with an incubation time of 24 h. The quantity and the diffusion area of the cells in 1Ce/1Gd@HA/PLGA and 1Ce@HA/PLGA groups are larger than those in HA/PLGA, 0.5Gd@HA/PLGA, and 1Gd@HA/PLGA groups, probably resulting from the higher Ce concentration in 1Ce/1Gd@HA/PLGA and 1Ce@HA/PLGA groups (John et al., 2003). With equal Ce^3+^ doping concentration, the results of the Ce^3+^ doping group are similar to those of Ce/Gd co-doping groups, indicating that the results are not involved by Gd doping. The aforementioned results demonstrate that the doping of Ce^3+^ promotes cell diffusion and communication among cells and enhances cell proliferation and adhesion on the HA medium.

### Osteogenic Differentiation

**FIGURE 8:**
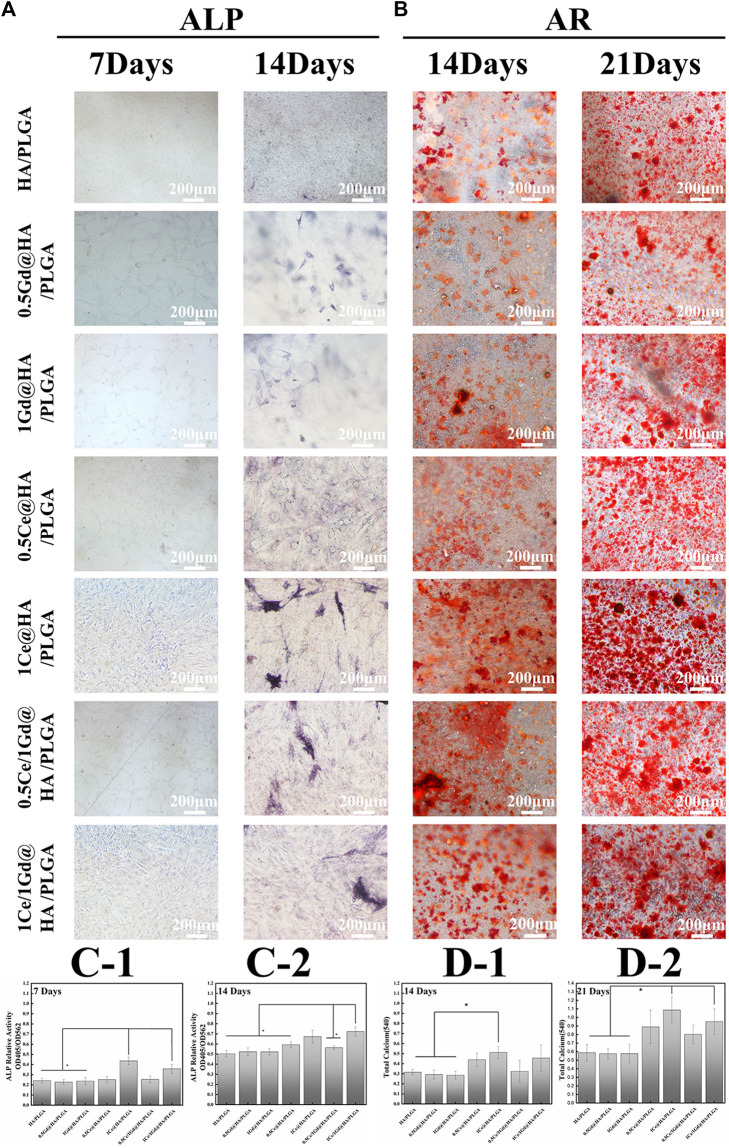
MC3T3-E1 cells stained by **(A)** ALP and **(B)** AR cultured for 7 and 14 days and 14 and 21 days using different nanocomposites. Scale bar, 200 μm. **(C1-2)** ALP activity of MC3T3-E1 cells cultured for 7 and 14 days was quantitatively evaluated using different nanocomposites. **(D1-2)** Different nanocomposites were used to quantitatively evaluate the calcium content and mineral deposition of MC3T3-E1 cells which was cultured for 14 and 21 days.* *p* < 0.05.

Alizarin red and alkaline phosphatase (ALP) were used in the osteogenic differentiation processes of MC3T3-E1 cells on the scaffolds with different nanocomposites. ALP, a marker for osteoblast differentiation, is critical for early osteoblast differentiation (Rosales-Rocabado et al., 2014). The high expression of ALP represents the enhanced osteogenic capacity of the cells (Gaharwar et al., 2013). Figure 8A shows the optical microscopy images of the MC3T3-E1 cells after ALP on the 7^th^ and 14^th^ days during cell culture, which illustrate that obvious ALP positive staining occurred after 7 days for 1Ce@HA/PLGA and 1Ce/1Gd@HA/PLGA groups (Figures 8C–1), while this phenomenon did not appear in the other groups, indicating that the osteoblast differentiation in the early and middle stages depends on Ce^3+^ concentration. On the 14^th^ day, ALP staining is enhanced in all the groups. However, ALP staining is slightly more decreased for HA/PLGA, 0.5Gd@HA/PLGA, and 1Gd@HA/PLGA groups than that for the other groups (Figures 8C–2). This indicates that Ce could enhance the osteoblast differentiation process. The activity of ALP in the 1Ce/1Gd@HA/PLGA group is the highest among all the groups, showing a statistical difference (*p* < 0.05). This is different from cell adhesion results. The release of Ce^3+^ may reach a peak with the extension of time, and the effect on cells does not change with the increase in concentration (Yang et al., 2021). ALP staining results demonstrate that cell proliferation and adhesion are enhanced on HA/PLGA composites doped with Ce^3+^, and the ability of *in vitro* osteogenic induction is also improved.

Cellular calcium deposition is also a commonly used indicator for osteogenic differentiation. In this study, various scaffolds were used to culture MC3T3-E1 cells for 2 and 3 weeks, and then quantitatively, the formation of calcium nodules was analyzed by alizarin red S staining. As shown in Figure 8B, a few calcium nodules occurred on the cell surface on the 14^th^ day, while large amounts of calcium nodules generated on the 21^st^ day. Both the staining intensity and staining area significantly increased on the 21^st^ day. With the same incubation time, both the staining intensity and size of calcium nodules in the Ce-doped groups are larger than those in the other groups. The staining intensity in 1Ce@HA/PLGA and 1Ce/1Gd@HA/PLGA groups is higher than that in the other groups. This indicates that Ce doping can promote the maturation of osteoblastic cells with a concentration-dependent effect.

According to the quantitative analysis (Figures 8D1–D2), the calcium content in HA/PLGA and Gd@HA/PLGA groups is obviously lower than that in 1Ce@HA/PLGA group on the 14^th^ day, while on the 21^st^ day, the calcium content of the former significantly improved, demonstrating that the Ce doping into scaffolds could enhance the calcium deposition and osteoblastic induction ability.

### Expression of Osteogenesis-Related Proteins

To investigate the osteogenic differentiation process of Ce/Gd@HA/PLGA nanocomposites, osteogenic markers (osteocalcin, OCN) and bone morphogenic protein (type I collagen, COL-1) were immunofluorescence stained. OCN, an important marker of bone formation, is a protein expressed during osteoblast maturation (Chou et al., 2005). COL-1 is a channel protein shared by multiple pathways, covering the entire osteogenesis process. For bone mineralization, both the aforementioned proteins are essential, and they are also widely expressed during the osteogenesis process (Liu et al., 2013).

**FIGURE 9:**
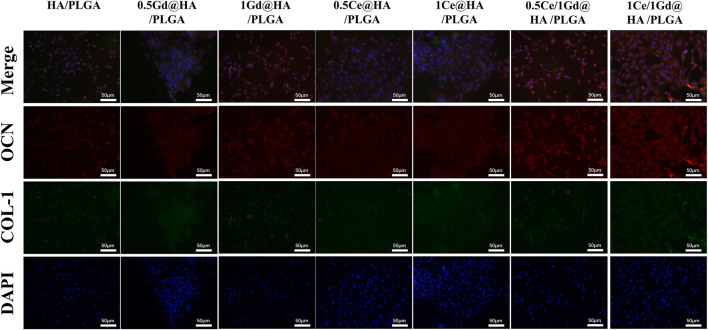
Osteogenic markers OCN and COL-1 immunofluorescence labeling of cells cultured on different substrates for 14 days. Scale bar, 50 μm.

As shown in Figure 9, the number of cells in Ce-doped group is larger than in other groups. As the doping concentration of Ce increases, the immunofluorescence intensity in 1Ce@HA/PLGA and 1Ce/1Gd@HA/PLGA groups is much higher than that in other groups. The expression intensity of OCN and COL-1 in the 1Ce/1Gd@HA/PLGA group is the highest among all the groups, which almost covers the entire field of vision under the microscope. This further demonstrates that the expression of osteogenic proteins could be promoted by Ce^3+^ doping. A larger Ce^3+^ doping concentration correlates to a higher effect on the expression of osteogenic proteins. Yin’s research indicates that Ce ions have greatly increased the activity of osteogenic transcription factors (Runx2, Satb2, and OCN). Additionally, the expression of bone morphogenetic protein-2 (BMP-2) was enhanced by Smad1/5/8, a key signal pathway during osteogenesis. According to the experimental results, the osteogenic differentiation process could not be affected by Gd^3+^ doping.

The proliferation, adhesion, and osteogenic differentiation of MC3T3-E1 cells in 1Ce/1Gd@HA/PLGA and 1Ce@HA/PLGA groups could be promoted, according to the aforementioned *in vitro* experimental results.

### Animal Studies

**FIGURE 10:**
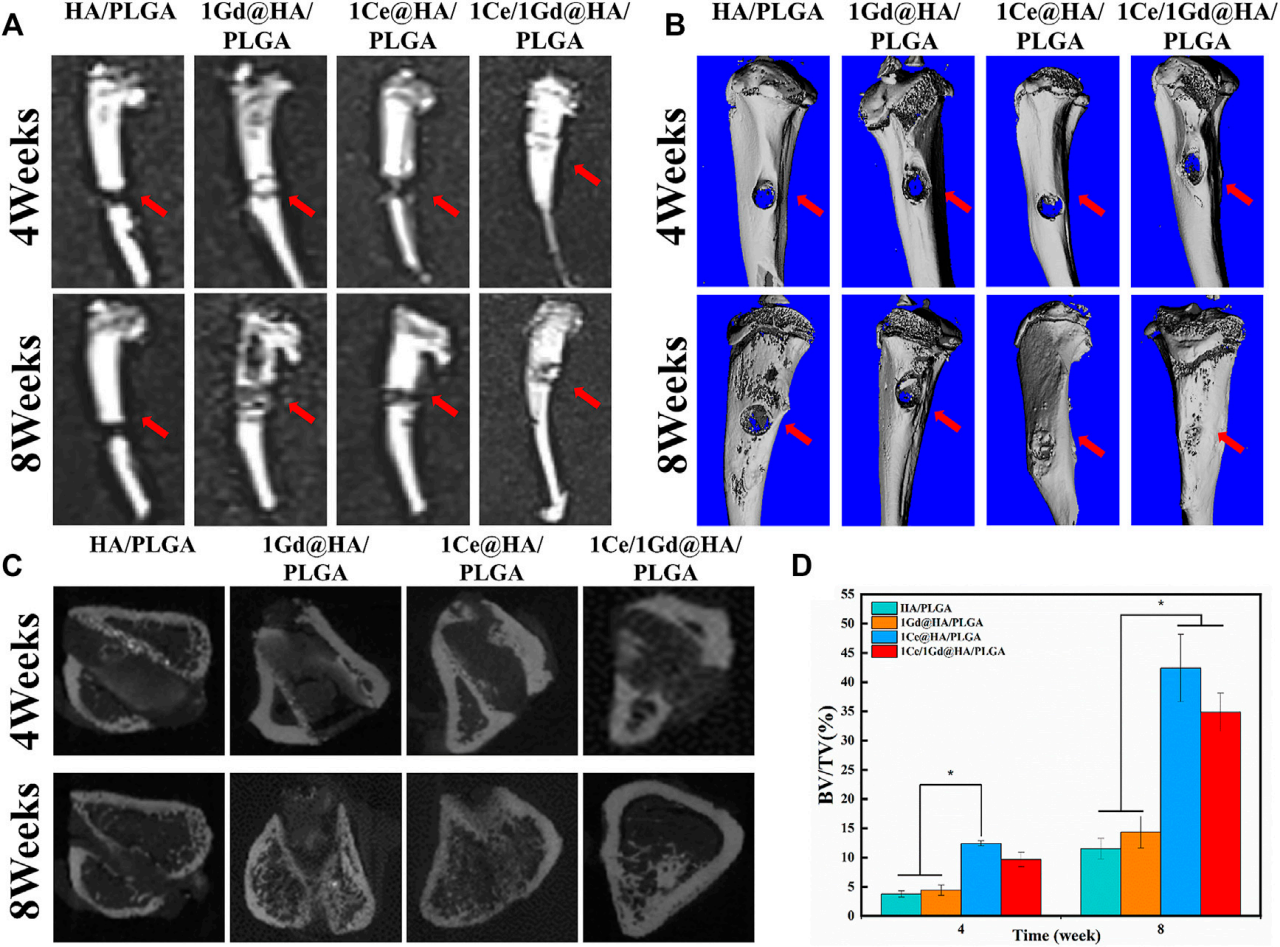
**(A)** T1 MRI images of rats after 4 and 8 weeks of operation. **(B)** Three-dimensional and **(C)** sagittal planes of the rat new bone were reconstructed at 4 and 8 weeks after operation, and **(D)** the regenerated bone bv/TV was quantitatively analyzed by HA/PLGA, 1Ce@HA/PLGA, 1Gd@HA/PLGA, and 1Ce/1Gd@HA/PLGA scaffolds. The red arrow indicates the location of the bone defect. * *p* < 0.05.

Figure 10A shows T1 MRI images of the bone defects in the 4^th^ and 8^th^ weeks after rat tibial implantation with HA/PLGA, 1Ce@HA/PLGA, 1Gd@HA/PLGA, and 1Ce/1Gd@HA/PLGA scaffolds. In the 4^th^ week, HA/PLGA and 1Ce@HA/PLGA composites were not observed, while we can clearly observe an enhanced MRI signal in 1Gd@HA/PLGA and 1Ce/1Gd@HA/PLGA composites. In the 8^th^ week, the brightness of 1Gd@HA/PLGA and 1Ce/1Gd@HA/PLGA composites was reduced compared with that in the 4^th^ week, due to the decrease in the Gd^3+^ concentration caused by the degradation of the scaffolds or the release of nanoparticles (Huang et al., 2016).

Micro-CT reconstructions were carried out for the bilateral tibia of the rats in the 4^th^ and 8^th^ weeks. As shown in Figure 10B, the new bones were not formed among all the groups in the 4th week. It indicates that bone tissue repair and reconstruction were not obvious within 4 weeks. After 8 weeks, bone defects were significantly repaired, a mass of poroma formed, and the surrounding bone tissue began to grow into the defect area. For 1Ce/1Gd@HA/PLGA and 1Ce@HA/PLGA groups, the bone defect areas were covered by new bone tissues. Figure 10C shows a CT transverse view of the bone defects. At the 4^th^ and 8^th^ weeks, the bone mass in HA/PLGA and 1Gd@HA/PLGA groups was nearly kept unchanged. Abundant tropical generated and bone tissue grew significantly for 1Ce@HA/PLGA and 1Ce/1Gd@HA/PLGA groups, which is consistent with the images of 3D surface reconstruction. The values of BV/TV increased with time (Figure 10D).

The results obtained from the aforementioned studies demonstrate that HA/PLGA scaffolds doped with Ce^3+^ could effectively promote new bone formation. We could clearly observe the Ce/Gd@HA/PLGA scaffolds in MRI images. MRI capability is enhanced by Gd^3+^ doping without affecting the bone formation process.

### Histological Analysis

**FIGURE 11:**
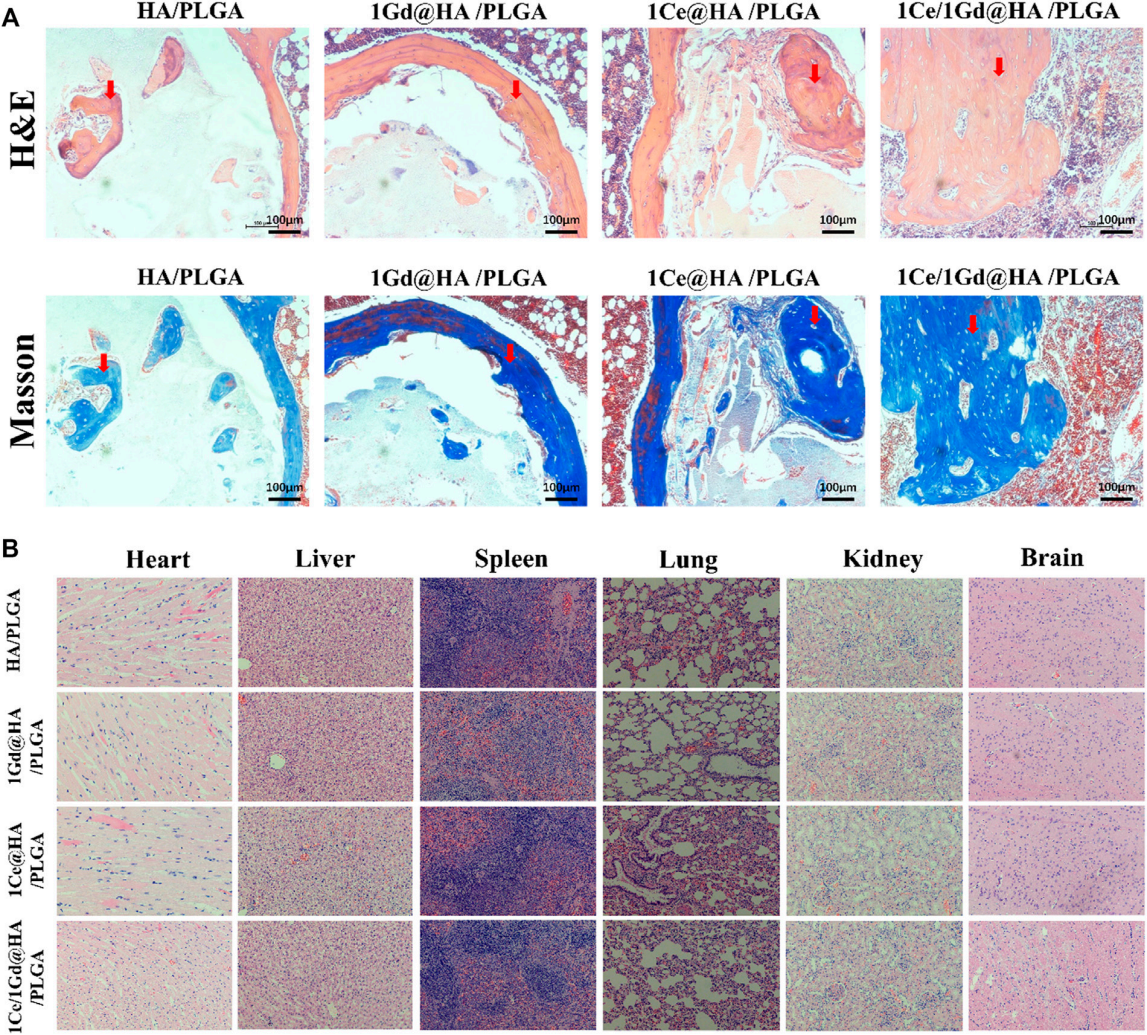
**(A)** H&E and Masson trichrome staining of cross sections of tibial defects in rats after 8-week implantation. Scale bar, 100 μm. Red arrows indicate newly formed bone. **(B)** H&E staining of rat’s heart, liver, spleen, lung, kidney, and brain 8 weeks after implantation.

Hematoxylin and eosin (H&E) and Masson staining were used for histological analysis to evaluate the histological repair of tibial defects at the 8^th^ week. From Figure 11A, one can see that the scaffolds are located in the defect area without obvious movement. No significant inflammation was found in all groups. The scaffolds could be used as frame structures for new bone penetration. A handful of new bone tissues was formed near the defect interface, with only little fibrous tissue inside the scaffolds, for HA/PLGA and 1Gd@HA/PLGA groups. However, for 1Ce@HA/PLGA and 1Ce/1Gd@HA/PLGA groups, new bone penetration accrued, and numerous mature bone tissues and myeloid tissues could be observed. Granulation tissues and fibrous scabs mixed with the nanocomposites could be observed in the scaffolds.

### *In Vivo* Biosafety Tests

At the 8^th^ week, H&E staining was used to observe the effects of cerium- and gadolinium-implanted stents on rat heart, liver, spleen, lung, brain, and kidney tissues. The H&E staining results demonstrate that no abnormal or pathological changes occurred to the rats in all groups (Figure 11B).

## Conclusion

In the present study, Ce/Gd co-doped HA nanoparticles were successfully synthesized by using the hydrothermal synthesis method and Ce/Gd@HA NPS with typical hexagonal crystal structures. Ce/Gd@HA/PLGA scaffolds enhanced MRI images due to the doping of Gd^3+^, providing a feasible way to observe implants non-invasively and non-radioactively. The doping of Ce^3+^ reduced the effect of excessive Gd^3+^ doping on HA crystal structures, without affecting the MRI capacity. *In vitro* experiments demonstrated that 1Ce/1Gd@HA/PLGA scaffolds could promote the process of cell adhesion, proliferation, and osteogenic differentiation. Better osteoinduction capacity occurs in 1Ce/1Gd@HA/PLGA and 1Ce@HA/PLGA groups than that in other groups.

The *in vivo* experiments indicated that the implanted scaffolds in HA/PLGA and 1Ce@HA/PLGA groups could not be observed in the 4^th^ week, while the implanted scaffolds can be clearly observed in 1Ce/1Gd@HA/PLGA and 1Gd@HA/PLGA groups. The results of micro-CT and tissue staining showed that the implanted 1Ce/1Gd@HA/PLGA scaffold had significant osteogenesis at the 8^th^ week.

1Ce/1Gd@HA/PLGA composites could both promote the bone repair process and exhibit MRI ability, which could be used to confirm successful implantation. All aforementioned results demonstrate that 1Ce/1Gd@HA/PLGA composite will be an attractive tissue engineering material for future bone defect repair.

## Data Availability

The original contributions presented in the study are included in the article/Supplementary Material, further inquiries can be directed to the corresponding authors.
