# Supplementary figures and images for: Synthesis of Ce/Gd@HA/PLGA Scaffolds Contributing to Bone Repair and MRI Enhancement
Source: Front Bioeng Biotechnol. 2022 Mar 31;10:834226. doi: 10.3389/fbioe.2022.834226 (PMC9009416; doi:10.3389/fbioe.2022.834226)

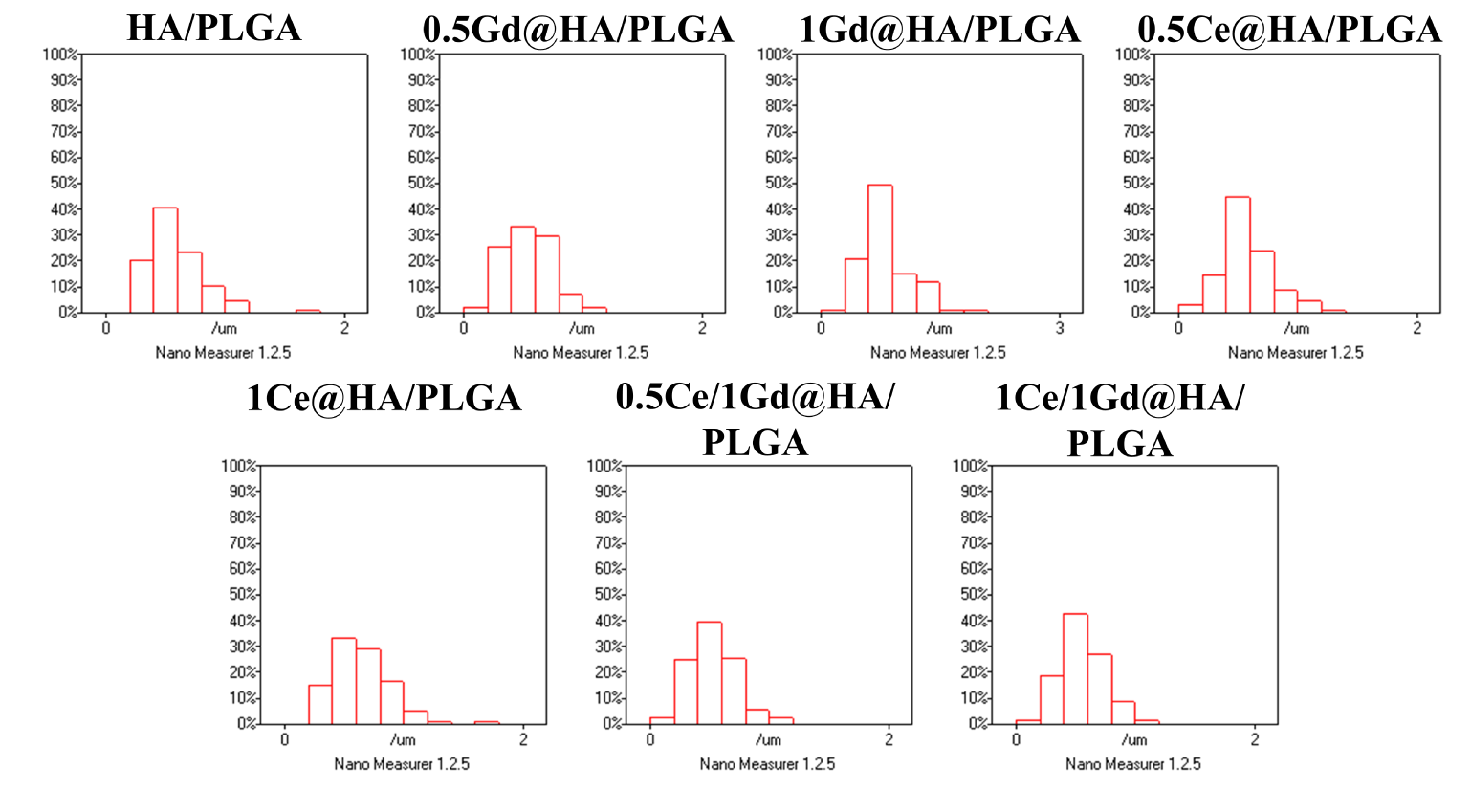

Supplement: Supplementary file 2 [file Image1.TIF]
